# A Multiscale Investigation of Bicoid-Dependent Transcriptional Events in *Drosophila* Embryos

**DOI:** 10.1371/journal.pone.0019122

**Published:** 2011-04-22

**Authors:** Feng He, Jie Ren, Wei Wang, Jun Ma

**Affiliations:** 1 Division of Biomedical Informatics, Cincinnati Children's Research Foundation, Cincinnati, Ohio, United States of America; 2 Division of Developmental Biology, Cincinnati Children's Research Foundation, Cincinnati, Ohio, United States of America; 3 Key Laboratory of Cell Proliferation and Differentiation, Center of Developmental Biology and Genetics, College of Life Sciences, Peking University, Beijing, People's Republic of China; Skirball Institute of Biomolecular Medicine - New York University Medical Center, United States of America

## Abstract

**Background:**

Morphogen molecules form concentration gradients to provide spatial information to cells in a developing embryo. Precisely how cells decode such information to form patterns with sharp boundaries remains an open question. For example, it remains controversial whether the *Drosophila* morphogenetic protein Bicoid (Bcd) plays a transient or sustained role in activating its target genes to establish sharp expression boundaries during development.

**Methodology/Principal Findings:**

In this study, we describe a method to simultaneously detect Bcd and the nascent transcripts of its target genes in developing embryos. This method allows us to investigate the relationship between Bcd and the transcriptional status of individual copies of its target genes on distinct scales. We show that, on three scales analyzed concurrently—embryonic, nuclear and local, the actively-transcribing gene copies are associated with high Bcd concentrations. These results underscore the importance of Bcd as a sustained input for transcriptional decisions of individual copies of its target genes during development. We also show that the Bcd-dependent transcriptional decisions have a significantly higher noise than Bcd-dependent gene products, suggesting that, consistent with theoretical studies, time and/or space averaging reduces the noise of Bcd-activated transcriptional output. Finally, our analysis of an X-linked Bcd target gene reveals that Bcd-dependent transcription bursts at twice the frequency in males as in females, providing a mechanism for dosage compensation in early *Drosophila* embryos.

**Conclusion/Significance:**

Our study represents a first experimental uncovering of the actions of Bcd in controlling the actual transcriptional events while its positional information is decoded during development. It establishes a sustained role of Bcd in transcriptional decisions of individual copies of its target genes to generate sharp expression boundaries. It also provides an experimental evaluation of the effect of time and/or space averaging on Bcd-dependent transcriptional output, and establishes a dosage compensation mechanism in early *Drosophila* embryos.

## Introduction

Regulation of transcription plays a pivotal role in many biological processes including pattern formation during embryonic development [1], [2], [3], [4]. While transcription is a time-evolving process inside a cell, understanding how its regulation participates in the decoding of spatial information in a developmental system requires a concurrent consideration of time and space on a much larger scale. It is thus necessary to integrate the events that take place on distinct time and space scales, ranging from chemical reactions inside a cell to the coding and decoding of spatial information in an entire embryo or tissue. In this study, we perform a multiscale experimental investigation of the relationship between the input of the Bicoid (Bcd) activator and its target gene responses during development.

Bcd is a morphogenetic protein that forms a concentration gradient along the anterior-posterior (A-P) axis in early *Drosophila* embryos [5], [6], [7], [8]. While the Bcd gradient profile is relatively smooth following an exponential function along the A-P axis, its target genes respond to this concentration gradient input to achieve expression patterns with relatively sharp boundaries [9], [10], [11], [12], [13]. Since such on/off expression boundaries, which are common in developmental systems, play critical roles in instructing cell fates, understanding the precise mechanisms leading to these outcomes represents a fundamental problem in biology [14], [15], [16]. While most experimental studies performed up to date have focused on the input-output relationship between Bcd and its target genes on the embryonic scale through the analysis of mature mRNA or protein [9], [10], [11], [12], [13], [17], [18], [19], [20], [21], there is currently no knowledge about this relationship on time and space scales that are approaching the actual transcriptional events. Such information is essential for evaluating the controversial question of whether Bcd plays a transient or sustained role in the transcriptional decisions of its target genes to generate sharp expression boundaries during development.

Here we describe experiments to simultaneously detect Bcd protein and the nascent transcripts of its native target genes inside the nuclei of developing embryos. We generate large experimental datasets to probe concurrently the input-output relationship between Bcd and the transcriptional status of its target genes on three distinct scales, embryonic (i.e., along the A-P axis of the embryo), nuclear (i.e., at a given A-P position) and local (i.e., at the sites of nascent transcripts). Our results reveal an association between transcriptional burst events and Bcd concentrations on all three scales. These results underscore a sustained role of Bcd in the transcriptional decisions of its target genes as embryonic development progresses. We also provide experimental evidence suggesting that, consistent with theoretical studies [22], [23], [24], time and/or space averaging can reduce the noise of Bcd-dependent transcriptional output. Furthermore, our analysis of an X-linked Bcd target gene reveals important insights into dosage compensation mechanisms in early embryos [25], [26]. Our study provides an integrated, multiscale view of the relationship between Bcd and its target gene transcription, suggesting that this chemical reaction-driven relationship transcends its role in instructing A-P patterning.

## Results

### Experimental design

**Figure 1:**
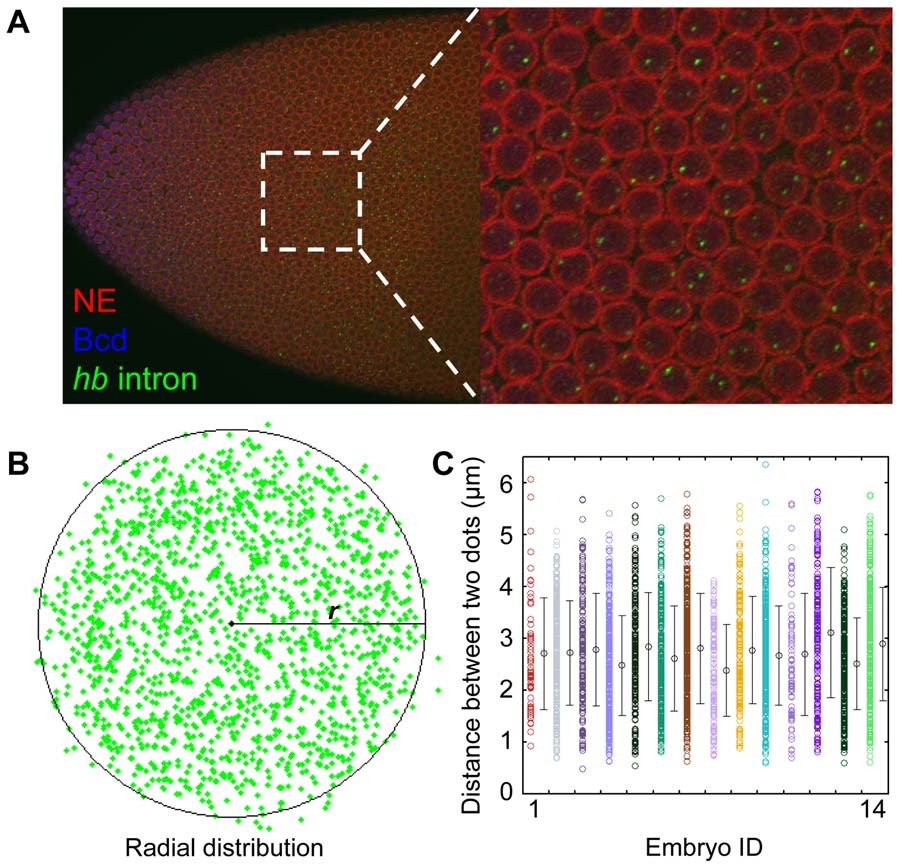
Simultaneous detection of Bcd and nascent *hb* transcripts in embryos. (A) Shown is a merged Confocal image of a wt embryo at early nuclear cycle 14 detecting the nuclear envelope (red), Bcd protein (blue) and the nascent *hb* transcripts as intron dots (green). Shown on the right is a magnified view of a section of the expression region. (B) Shown is the radial distribution of detected *hb* intron dots. Data shown here were extracted from one single wt embryo with 2,556 identified nuclei (with a mean diameter *l* = 6.06±0.80 µm) and 1,537 detected intron dots. Intron dots that appear to be outside the boundaries of this illustrative “average” nucleus are due to the fact that real nuclei are not all perfectly round in shape. As explained in the text, our intron dot dataset used in all the analyses described in this work were extracted from within the nuclei. (C) Shown are the measured distances between two detected *hb* intron dots inside individual nuclei. The measured mean distance between two intron dots inside individual nuclei is 2.70±0.18 µm for all 14 tested embryos (represented by different colors). Each error bar is one standard deviation among the nuclei for a single embryo.

We developed a method to simultaneously detect Bcd and the nascent transcripts of its target genes in early *Drosophila* embryos. This method combined fluorescence antibody staining to detect Bcd [12] with fluorescence *in situ* hybridization (FISH) using intronic probes to detect target genes' nascent transcripts [27]. The quantitative and simultaneous detection of Bcd in our method allows us to evaluate directly the relationship between the Bcd input and the nascent transcript output of its target genes at the level of individual gene copies. We focused on two Bcd target genes, *hunchback* (*hb*) and *orthodentical* (*otd*), for our study. Figure 1A shows a merged high-resolution Confocal image detecting Bcd protein (blue), the nascent transcripts of *hb* as distinct “intron dots” (green), and the nuclear envelope (red) in an embryo at early nuclear cycle 14. The nuclear marking allows us to extract data from within the nucleus, a location relevant to the input-output relationship in transcription. It also allows us to adjust our algorithms for detecting intron dots and to help estimate errors in such detections (see Materials and Methods and Figure S1). Figure 1B shows that *hb* intron dots are randomly distributed on the transverse plane but are constrained along the apical-basal axis [28], a property that enables our *z*-sections to capture all intron dots inside the interphase nuclei in flattened embryos (see Materials and Methods). In our analysis, the detected *hb* intron dots within an interphase nucleus are well separated from each other (Figure 1C), with a mean distance of 2.70±0.18 µm, suggesting that they are well resolved to allow the scoring of individual intron dots [29], [30]. A total of ∼13,000 *hb* intron dots were captured from the Bcd-dependent anterior expression regions of 14 wt embryos at early nuclear cycle 14. As shown in a recent study [31], embryos at nuclear cycle 14, unlike those at earlier stages, exhibit a more stochastic pattern in Bcd target gene transcription, a feature that makes this stage particularly useful for investigating the sustained role of Bcd in transcriptional decisions.

**Figure 2:**
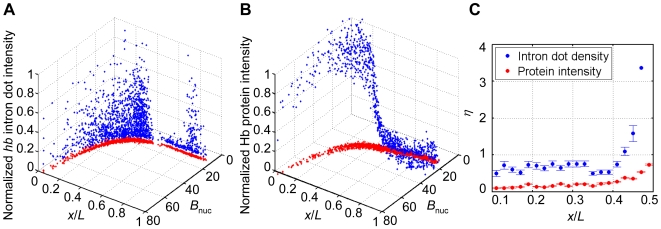
Expression profiles for *hb* intron dots and Hb proteins. (A) Shown is a 3-D scatter plot of normalized A-P positions (*x*/*L*), mean Bcd intensities of the nucleus (*B*_nuc_) and the normalized intensities of *hb* intronic mRNA within identified dots for a single embryo. The projection onto the *x*/*L*-*B*_nuc_ plane (red) gives the profile of the nuclear Bcd intensity as a function of the A-P position. (B) Shown is a 3-D scatter plot of normalized A-P positions (*x*/*L*), mean Bcd intensities of the nucleus (*B*_nuc_) and the normalized intensities of nuclear Hb protein for a single embryo. The projection onto the *x*/*L*-*B*_nuc_ plane (red) gives the profile of the nuclear Bcd intensity as a function of the A-P position. (C) Shown are the noise (*η*) profiles of *hb* intron dot density (*ρ*) for a single embryo (blue) and the noise for Hb protein intensity for a single embryo (red) as a function of *x*/*L*. Error bars were obtained from bootstrapping.

In addition to the *hb* intron dot data extracted from our Confocal images, we also measured simultaneously Bcd concentrations in embryos. As discussed previously [9], [12], [32], [33], the detected Bcd intensities in our analysis have a linear relationship with the number of Bcd molecules. Thus we used the mean pixel intensities for Bcd within a nucleus, *B*_nuc_ = 〈*B*_pix_〉, as the nuclear Bcd concentrations, where *B*_pix_ is the detected Bcd intensity value of each pixel inside the nucleus. Figure 2A is a 3-D scatter plot exhibiting the normalized A-P positions (*x*/*L*), the corresponding *B*_nuc_, and normalized intensities of identified *hb* intron dots for all the captured nuclei from our experimental embryos.

### *hb* intron dot density exhibits a Hill function in response to Bcd along the A-P axis

**Figure 3:**
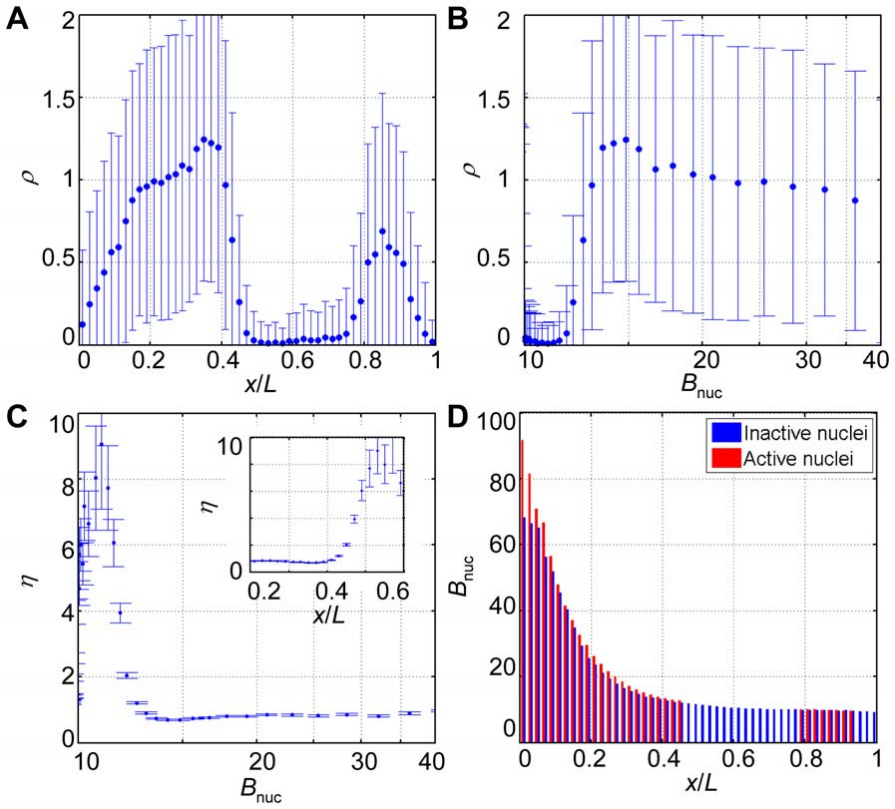
Investigating the role of Bcd in *hb* transcriptional events on embryonic and nuclear scales. (A) Shown is the number density of *hb* intron dots (*ρ*) as a function of the A-P position (*x*/*L*). An error bar is one standard deviation. (B) Shown is the profile of *ρ* as a function of mean *B*_nuc_ (on a log scale) of different bins along the A-P axis. An error bar is one standard deviation. (C) Shown is the measured noise *η* as a function of either mean *B*_nuc_ (on a log scale) of different bins or the bin position *x*/*L* (inset). Error bars were obtained from bootstrapping. (D) Shown are the mean Bcd intensities within the inactive nuclei (blue) and the active nuclei (red) at different A-P positions. For the anterior *hb* expression region (*x*/*L* = 0.16∼0.46), where *hb* expression is dependent on Bcd concentration, *p*-values<0.0001 (Student's t-tests); at the posterior of the embryos (*x*/*L* = 0.82∼0.96), where a Bcd-independent *hb* expression domain is detected, *p*-values>0.1; for the non-expressing regions of the embryos (*x*/*L* = 0.46∼0.82 and 0.96∼1), where the burst probability is too low (<0.05) to allow reliable computation of the mean Bcd intensities within the active nuclei, only *B*_nuc_ within inactive nuclei are shown.

We define the number density of *hb* intron dots within a bin of nuclei along the A-P axis as *ρ* = *n*/*N*, where *n* is the number of detected intron dots and *N* is the number of nuclei. To quantify the input-output relationship between the nuclear Bcd concentration and the nascent *hb* transcripts on the embryonic scale, we plot the number density of *hb* intron dots *ρ* either as a function of the normalized A-P position (*x*/*L*) (Figure 3A) or as a function of the averaged nuclear Bcd concentrations (*B*_nuc_) of the bins along the A-P axis (Figure 3B). The intron dot density *ρ* profile as a function of A-P position (Figure 3A) resembles broadly the profiles of Hb proteins at a comparable developmental time [9], [10], [11], [12], [19], [32], [33] (see below for a quantitative comparison). Figure 3B shows that the *hb* intron dot density profile exhibits a characteristic Hill-like response to the nuclear Bcd input *B*_nuc_ [11], [12]. The Hill-like input-output relationship between Bcd and *hb* nascent transcripts on the embryonic scale indicates that the mean transcriptional status of a population of individual copies of the *hb* gene in embryos responds to the graded input of nuclear Bcd concentrations in a highly cooperative manner resembling the DNA binding properties of Bcd measured biochemically [34], [35].

### Properties of transcriptional status and transcriptional products reveal effects of time and/or space averaging

To investigate the relationship between gene transcriptional status and transcriptional products in response to the Bcd gradient in embryos, we compared our *hb* intron dot data with Hb protein data, both obtained from flattened embryos at a comparable developmental time. Figure 2B shows Hb protein intensities as a function of *B*_nuc_ and *x*/*L* in a 3-D scatter plot (see Figure 2A for an equivalent plot of *hb* intron data). In our analysis, we evaluated the following three specific properties that describe the target gene responses to the Bcd gradient: the boundary positions detected as either the intron dot density or nuclear Hb protein intensities, their respective Hill coefficient values and the noise levels.

Our comparative analysis reveals two results. First, the detected intron dot density boundary position (the A-P position *x*/*L* where *ρ* is half maximal), 0.43±0.02 (standard deviation calculated from 14 embryos), is comparable to the boundary position detected by Hb protein, 0.44±0.01 (calculated from 5 embryos). Second and importantly, the *ρ* profiles (Figures 3A and B) extracted from the intron dot data are both sharper and noisier than the Hb protein intensity profiles. Specifically, while the Hill coefficient value calculated from the intron dot data is 6.1±2.6 (calculated from 14 embryos), this value for the Hb protein profiles is 5.2±0.4 (calculated from 5 embryos). In addition, while the noise of the *ρ* profiles (*η* = *σ*/*ρ*) is ∼80% at the anterior expression regions (Figure 3C), the Hb protein profiles had a noise of less than 20%. Data extracted from single embryos exhibit similar noise differences (Figure 2C). The higher noise of *hb* intron dots relative to the Hb protein intensities is also reflected by the variations of the detected boundary positions (2% vs. 1% embryo length, respectively). Recent theoretical studies suggest that averaging of target gene products over space and/or time can reduce the noise of transcriptional responses of Bcd target genes [22], [23], [24]. Our comparative analysis provides a direct experimental demonstration of this hypothesis. Our results also provide experimental support to a theoretical prediction that averaging over space, while capable of reducing target expression noise, also reduces the steepness of target gene responses (i.e., a reduction of the effective Hill coefficient) [23], [24].

### The effect of fluctuations in nuclear Bcd concentrations on *hb* transcriptional status

To further evaluate the role of Bcd in transcriptional decisions of its target genes, we analyzed properties of the nuclei within individual bins along the A-P axis. While the differences in the average nuclear Bcd concentrations between bins along the A-P axis encode spatial information on the embryonic scales (as discussed above), differences in Bcd concentrations of individual nuclei within the bins represent noise that is encountered by the system. To determine whether such Bcd concentration noise may be propagated to the transcriptional status of its target genes, we compared the nuclei (within bins) that either have or lack intron dots, referred to as active or inactive nuclei, respectively. Figure 3D shows that, on average, *B*_nuc_ values are significantly higher in the active nuclei than those in the inactive nuclei at the anterior expression region (see its legend for details). An analysis using data from individual embryos, thus eliminating between-embryo fluctuations in nuclear Bcd concentrations, revealed similar results (Figure S2). Together, these results show that, on the nuclear scale, fluctuations in nuclear Bcd concentrations (within a bin) that do not encode spatial information along the A-P axis are also propagated to *hb* transcriptional status. These results show that Bcd activates transcription of its target genes in a manner (i.e., following an input-output relationship) that transcends its morphogenetic role in instructing A-P patterning (Figure 2B). They demonstrate that Bcd is a sustained input for the transcriptional decisions of individual copies its target gene *hb* at the developmental time of our analysis.

### The detected intron dot sites exhibit an enrichment of Bcd molecules

**Figure 4:**
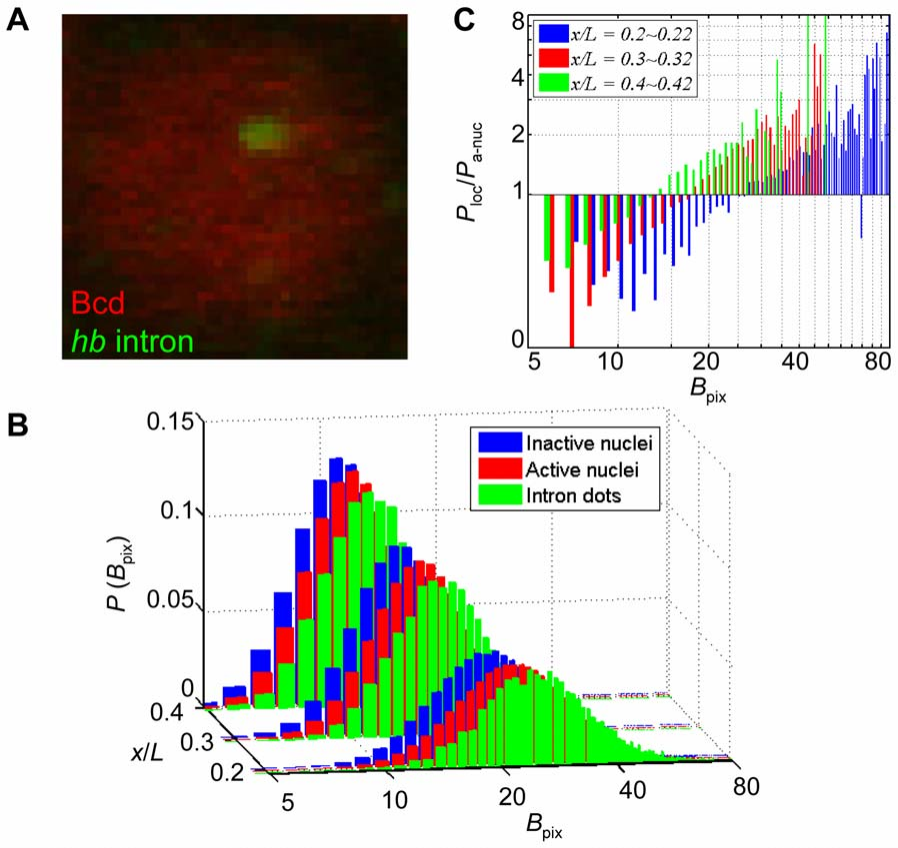
Investigating the role of Bcd in *hb* transcriptional events on the local scale. (A) A zoom-in merged view of a nucleus showing a single detected *hb* intron dot (green) and Bcd intensities (red). (B) Shown are the probability density functions of Bcd pixel intensity, *P*(*B*_pix_), inside the inactive nuclei (blue), the active nuclei (red), or the areas of the detected intron dot sites (green). Data from three different A-P positions are shown in different colors. (C) Ratio of *P*(*B*_pix_) inside the areas of the detected intron dot sites to *P*(*B*_pix_) within the active nuclei. A base line at ratio of 1 is drawn (see text for details). Different colors represent data at different A-P positions: *x*/*L* = 0.2∼0.22 (blue), *x*/*L* = 0.3∼0.32 (red) and *x*/*L* = 0.4∼0.42 (green).

While the diffusion constant of Bcd remains highly controversial, independent experiments support the existence of a specie of Bcd molecules that have a limited mobility inside the nucleus, with a diffusion coefficient in the order of ∼0.3 µm^2^ s^−1^ [31], [33], [36]. The existence of a low-mobility species is consistent with the suggestion that transcription factors may become transiently immobilized when they frequently encounter and interact non-specifically with DNA [37]. This low-mobility species effectively makes the nucleus a system that is not well stirred: like many other transcription factors [38], [39], the native Bcd protein molecules are not evenly distributed within the nuclei of our experimental embryos (Figure 4A red). Previous high-resolution live-imaging studies have also revealed a similar punctate pattern of the distributions of Bcd molecules inside the nucleus [40].

To extend our investigation of the sustained role of Bcd in transcriptional decisions of its target genes, we analyzed Bcd intensities at the detected intron dot sites. As a transcriptional activator, Bcd molecules are expected to bind specific DNA sites in target gene enhancers and take residence there for a period of time necessary for eliciting functional responses. We sought to ask a simple question that can further determine whether Bcd has a direct and causal relationship with the transcriptional status of its target genes in embryos: are the local Bcd concentrations (i.e., the measured Bcd intensities at the detected intron dot sites) different from their corresponding nuclear environment (i.e., *B*_nuc_)? For our analysis, we plot histograms (Figure 4B) of the probabilities of pixel intensity values *B*_pix_ inside the inactive nuclei (green), the active nuclei (blue), or the areas of the detected intron dot sites (red) at different A-P positions. These histogram data reveal two results that are consistent among different bins (*x*/*L*) in the anterior *hb* expression regions of the embryos. First, the peak locations of the probability density function (i.e., the modes) are higher for the active nuclei (22, 15 and 12 for bins at *x*/*L* = 0.2∼0.22, 0.3∼0.32 and 0.4∼0.42, respectively) than their inactive counterparts (20, 13 and 11 for same respective bins). The distributions of pixel intensities from the active and inactive nuclei are also distinguishable from each other (*p*-values<0.0001, Kolmogorov-Smirnov tests). These findings are simply another way of expressing the results already presented in Figure 3D, i.e., the mean *B*_nuc_ is different between the active and inactive nuclei at given A-P positions.

Our second result shown in Figure 4B reveals that the probability density functions of *B*_pix_ for the active nuclei and for the detected intron dot sites (mode = 24, 16 and 13 for the bins at *x*/*L* = 0.2∼0.22, 0.3∼0.32 and 0.4∼0.42, respectively) are distinguishable from each other (*p*-values<0.0001, K-S tests). If the detected intron dot sites were randomly located (with regard to *B*_pix_ values) inside the active nuclei, one would have expected these two profiles to be similar to each other given that the total number of pixels inside the areas of all the detected intron dots within individual bins is sufficiently large (∼10,000 pixels). To further evaluate this point, we plot the *B*_pix_ probability ratio of intron dot sites to active nuclei (Figure 4C). In this plot, we draw a base line at the ratio of 1; data points above or below this line thus indicate an enrichment or deficit, respectively, in the observed *B*_pix_ at the detected intron dot sites relative to the nuclear environment. Our results (Figure 4C) show that, while there is a deficit in low-valued *B*_pix_ data points, high-valued *B*_pix_ data points are markedly enriched. This is true for all the tested A-P positions in the anterior expression regions of the embryo. Together, these results show that intron dot sites are associated with local Bcd concentrations that are higher on average than their nuclear environment. They represent experimental evidence further demonstrating that, despite the possibility that additional factors may also participate in activating *hb* transcription [17], [18], [19], [20], [31], [41], [42], [43], Bcd remains a direct input for *hb* transcriptional decisions at the developmental time of our analysis.

### Analysis of *otd* reveals mechanisms of dosage compensation in early embryos

**Figure 5:**
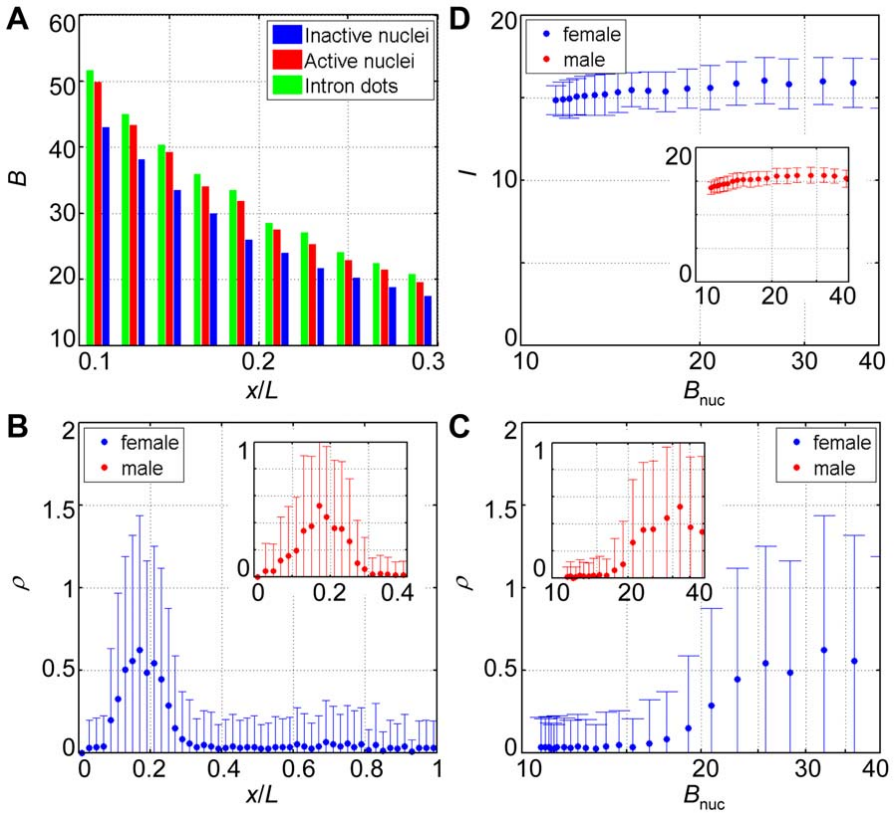
Multiscale investigation of the transcriptional properties of *otd*. (A) Shown are the mean Bcd intensities measured from the inactive nuclei (blue), the active nuclei (red) and the areas of the detected *otd* intron dots (green) as a function of *x*/*L*. Data were extracted from all 12 tested embryos (females and males). Student's t-tests were conducted to determine *p*-values for each A-P bin of nuclei. Comparing red with blue, all *p*-values<0.0001; comparing red with green, *p*-values are (in A-P order at the locations shown): 0.2, 0.1, 0.1, 0.01, 0.02, 0.06, 0.001, 0.02, 0.07 and 0.08. (B) Shown is the number density of *otd* intron dots (*ρ*) as a function of *x*/*L* for 8 female embryos (blue) or 4 male embryos (red, inset). An error bar is one standard deviation. (C) Shown is the number density of *otd* intron dots (*ρ*) as a function of mean *B*_nuc_ of different bins along the A-P axis for 8 female embryos (blue) or 4 male embryos (red, inset). An error bar is one standard deviation. Hill coefficient for the posterior boundary in response to the Bcd input is 4.0±1.9, corresponding to fewer binding sites for Bcd molecules in the *otd* enhancer than those in the *hb* enhancer [55]. (D) Shown is the raw intensity of *otd* intron dots (*I*) as a function of mean *B*_nuc_ of different bins along the A-P axis for 8 female embryos (blue) or 4 male embryos (red, inset). An error bar is one standard deviation.

To confirm and extend our findings based on *hb* transcription properties, we analyzed the profiles of the nascent transcripts of another Bcd target gene *otd* (see Figure S3). Our results reveal that *otd* transcription also exhibits a dependence on a sustained Bcd input. In particular, on the nuclear scale, the active nuclei (i.e., with at least 1 *otd* intron dot) have significantly higher *B*_nuc_ than their inactive counterparts on average (Figure 5A, compare red and blue). On the local scale, Bcd molecules are enriched at the detected intron dot sites (Figure 5A, compare red and green).

Since *otd* is located on the X chromosome, it allows us to investigate potential mechanisms of dosage compensation in early *Drosophila* embryos. In *Drosophila*, X-linked genes produce twice the amount of transcripts in males as in females, but the precise mechanisms of such up-regulation are not well understood [25], [26]. To investigate whether the *otd* transcriptional properties detected as intron dots may already exhibit distinct features in different sexes in embryos at early nuclear cycle 14, we separated embryos into females and males based on, respectively, the presence or absence of nuclei with two intron dots (for the inferred female and male embryos, the ratio of nuclei with two dots to nuclei with a single dot is 0.19±0.05 and 0.04±0.03, respectively; *p* = 0.0003, Student's *t* test). Our results show that, despite a two-fold difference in the copy number of *otd* in males and females, the *otd* intron dot density as a function of either *x*/*L* or *B*_nuc_ is approximately the same (Figures 5B and C). In addition, the mean intensities of *otd* intron dots do not exhibit significant differences between male and female embryos at given A-P positions (Figure 5D; *p*-values>0.1, Student's t-tests). These results suggest that, for *otd*, dosage compensation operative during embryogenesis allows this X-linked gene to burst in transcription at twice the probability in males as in females.

## Discussion

The simultaneous detection of both Bcd concentrations and the nascent transcripts of its target genes makes it possible to investigate the input-output relationship on three distinct scales in developing embryos. Our results show that copies of Bcd target genes respond to the activator input regardless of whether such an input encodes spatial information (i.e., as a *B*_nuc_ profile along the A-P axis) or not (i.e., as *B*_nuc_ fluctuations at given A-P positions). These results suggest that the input-output relationship between Bcd and its target gene transcription, a relationship governed by the contributing chemical reactions, transcends the morphogenetic role of Bcd in A-P patterning. A comparison between our *hb* intron dot data and Hb protein data reveals that both the boundary steepness and the expression precision exhibit marked differences (Figure 2). These results suggest that, in order for the system to gain the necessary expression precision and boundary steepness for the Hb protein (the gene expression product), it needs to produce a steeper profile in transcriptional decisions—that are noisier—for a group of individual *hb* gene copies. This delicate trade-off between precision and steepness is a consequence of averaging over space and/or time [23], [24].

Our large experimental datasets reveal that the Bcd intensities detected at the intron dot sites are higher than their nuclear environment (Figures 3D and 5D for both *hb* and *otd*). If Bcd does indeed act as a direct and sustained input for transcriptional decisions, one would have expected this property. Given a finite number of transcription factor molecules available inside a nucleus [11], an inevitable outcome of their binding to target gene enhancers is an enrichment of these molecules at the locations of their target genes [23]. In fact, through the use of activator fusion proteins and tandem repeats of reporter genes, the enrichment of activator molecules at their action sites is a well-documented phenomenon [44]. Importantly, our experimentally detected local enrichment is for native Bcd molecules at the actively-transcribing copies of native target genes in a native developmental system. Although binding of transcription factors to specific DNA can be highly-transient [44], our results suggest that the timescale of Bcd residence on the target gene enhancers likely approaches (or is comparable to) the timescale of mRNA splicing.

Recent studies show that the nuclear Bcd concentration profile is established as early as nuclear cycle 10 and it remains stable until nuclear cycle 14 [33]. Unlike the stable nuclear Bcd concentration profile, target gene expression patterns evolve significantly as a function of developmental time. For example, while the initial Hb expression boundary is relatively shallow, it becomes steeper as development progresses [13], [18], [31]. These results are consistent with previous suggestions that sharp expression boundaries are reflective of the contributions of feedback and cross-regulation mechanisms that are common in biology [17], [18], [45]. Our observed relationship between Bcd and its target gene transcription on both the nuclear and local scales at early nuclear cycle 14 demonstrates that Bcd remains a direct and relevant input for transcriptional decisions of its target genes at this stage of development. We suggest that the chemical reactions that govern the input-output relationship, in particularly cooperative binding [34], [35] of Bcd molecules to target enhancers inside the nucleus, contribute directly and persistently to the sharp expression boundaries of Bcd target genes on the embryonic scale. Recent studies show that, as development progresses, the patterns of *hb* transcriptional events exhibit a uniform-to-stochastic transition [31], which takes place concurrently as the maternal Hb protein begins to decay. This very nature of stochastic transcriptional bursts at nuclear cycle 14 makes it possible to specifically evaluate the input-output relationship on distinct scales that have uncovered a direct and sustained role of Bcd in *hb* transcription.

The results described in this report show that the probability of transcriptional bursts responds to the Bcd input in embryos. These results, together with those published recently [31], [46], are consistent with the hypothesis that activators play an important role in facilitating the inactive-to-active transition of promoter states. Such an activation mechanism is distinct from the activation mechanism for promoters that are already “pre-loaded” with RNA polymerase [46], [47]. We note that the overall intensities of individual intron dots of target genes exhibit significant variations that are independent of A-P positions or *B*_nuc_ (Figures 2A and 5D; see also [31]). These results suggest that developmental activators such as Bcd and maternal Hb, similar to activators in yeast cells [48], regulate primarily the burst frequency of transcription with little or no effect on burst size. One important finding presented in our current work is that the X-linked *otd* gene exhibits dosage compensation properties as early as nuclear cycle 14 during embryogenesis. Our results are consistent with a recent report that revealed widespread dosage compensation in syncytial embryos [49]. Together, these results raise an important question of precisely how dosage compensation may be achieved in such early embryos, since the MSL (male-specific lethal)-mediated system is not fully established until a later time [50], [51] (see [49] for a comprehensive discussion). While our current study does not specifically address this question, it does provide important insights into how *otd* transcription is regulated in male and female embryos. In particular, our results show that it is the probability of transcriptional bursts that is up-regulated in males, suggesting that X chromosomes in males possess properties that allow *otd* to burst in transcription at twice the frequency as in females. For X-linked genes whose transcription is not limited by the inactive-to-active transition of their promoter, dosage compensation likely regulates other steps in transcription [52]. We note that, although both maternal (as well as zygotic) Hb [31] and dosage compensation (Figure 5B) can affect the transcriptional burst frequency of Bcd target genes, they do not play a significant role in the expression boundary positions of these genes. These observations are consistent with our recent studies that evaluate experimentally and quantitatively the relationship between Bcd and the expression patterns of its target genes [12], [20], [53], [54]. Together, they further demonstrate that the positional information encoded by the Bcd gradient controls directly the positioning and precision of the expression boundaries of its target genes during development.

## Materials and Methods

### Probe preparation

Plasmids containing intronic sequences for *hb* and *otd* (the 2^nd^ and 3^rd^ intron, respectively) were constructed using PCR products amplified from *Drosophila* genomic DNA with the following primers: *hb* forward primer 5′-GCCATTACCAAGTGTCTCCATTTTG-3′; *hb* reverse primer 5′-AGAAAACAGAGAGAGTGGGGTTAGT-3′; *otd* forward primer 5′-AGATGTGCCATCCAGAGTACTAACT-3′; and *otd* reverse primer 5′-GAGGGCATTTCGCCTAATTAAACCA-3′. Both intronic probes were labeled with digoxigenin-11-dUTP (Roche Applied Sciences) and synthesized using the MAXIscript *In vitro* Transcription Kit (Ambion) according to manufacturer's instructions.

### FISH combined with protein staining

FISH combined with protein staining on whole-mount embryos was performed as follows. 1–4 hour *w^1118^* embryos collected at 25°C were fixed, rehydrated in a stepwise manner to PBST (0.137M NaCl, 0.003M KCl, 0.004M Na_2_HPO_4_, 0.002M KH_2_PO_4_, 0.1% Tween 20, pH7.4), post-fixed in 10% formaldehyde for 20 minutes, and washed in the Hybridization buffer B (50% formamide, 5× SSC, 0.1% Tween 20, 0.3% SDS) after being washed first in PBST. Embryos were then pre-hybridized for 1 hour in the Hybridization buffer A (50% formamide, 5× SSC, 0.1% Tween 20, 0.3% SDS, 50 µg/ml heparin, 10 µg/ml yeast tRNA, 100 µg/ml salmon sperm DNA) at 60°C, followed by hybridization overnight at 60°C in pre-warmed Hybridization buffer A containing the probes. Hybridized embryos were washed in Hybridization buffer B at 60°C, followed by washing 5 times in PBST at room temperature and incubation in Roche blocking buffer for 30 minutes. Embryos were incubated overnight at 4°C in Roche blocking buffer containing mouse anti-digoxigenin monoclonal primary antibody (Roche Applied Sciences; 1∶400 dilution) and rabbit anti-Bcd antibody (Santa Cruz Biotechnology; 1∶200 dilution), both of which had been presorbed. They were then washed in PBST 5 times at room temperature, incubated in PBST with Roche blocking buffer for 30 minutes, followed by incubation in Roche blocking buffer with Alexa Fluor 488-goat-anti-mouse secondary antibody (Invitrogen; 1∶400 dilution) and Alexa Fluor 647-goat-anti-rabbit secondary antibody (Invitrogen; 1∶400 dilution) for 1 hour at room temperature. Nuclear membrane was stained with Alexa Fluor 555-conjugated wheat germ agglutinin (Invitrogen; 1∶500 dilution) for 30 minutes at room temperature. After washing in PBST, embryos were mounted, without bridge, in ProLong® Gold antifade reagent (Invitrogen) for Confocal imaging.

### Confocal imaging

Embryos were imaged on an inverted Zeiss LSM 510 Confocal microscope with plan-neofluar 40×1.3 oil objective. Embryos at early nuclear cycle 14 were chosen to be imaged according to the nuclear density and shape. We imaged embryos that were “flattened” to maximize the number of nuclei that could be captured by Confocal *z*-sections for each embryo. Pinhole size was set such that the optical slice was 1 µm for all channels. The detector gain for each channel was set by, using an embryo with the characteristic expression pattern, adjusting the gain until only several pixels were saturated. Amplifier offset was adjusted for each channel so that pixels away from the embryo were nearly zero. In our experiment, the imaging of different embryos did not require adjustments of the setting between embryos or between slides; thus different embryos analyzed in our study have the same conversion factors between the captured fluorescence intensities and the number of detected molecules. In our analysis, we used flattened embryos and captured on average 6 *z*-sections for each region of an embryo taken in 0.5 µm intervals, which effectively captured all the intron dots in each nucleus. The images were 8-bit (with an intensity value ranging from 0 to 255) in depth and with 2048×2048 resolution (i.e., 160 nm/pixel). The captured *z*-stack images were projected and stitched together to generate the image data files of whole embryos for further analysis.

### Establishing threshold for intron dot detection

We used an integrated threshold setting that considered both the size and intensity for detecting intron dots, i.e., we defined an intron dot as a cluster of pixels that are above both a pixel intensity threshold and a pixel number threshold. We made the following considerations when setting the threshold for detecting intron dots. According to a general rule discussed previously [46], a threshold that is either too high or too low would prevent the detection of any expression patterns of the nascent transcripts (i.e., intron dots) in embryos. In our study, the threshold was chosen to maximize the total number of nuclear intron dots within the expression regions. We reason that a “true” expression pattern should possess certain characteristics that should be insensitive to changes in threshold setting (within a certain range). To specifically evaluate this point, we plot the difference in *hb* intron dot density (*ρ*) between two expression regions of the embryo (30%∼40% and 20%∼30% of embryo length) as a function of the pixel intensity threshold setting (while keeping the pixel number threshold at 4; Figure S1A). This plot shows that a plateau is reached at the intensity threshold of 32 (dashed line) and above, indicating that this threshold setting allows the detection of a “stable” profile while maximizing the total number of nuclear intron dots. Throughout the entire work, we used 4 as the pixel number threshold and 32 as the pixel intensity threshold. This threshold setting also satisfies the following three additional criteria: 1) to minimize the number density of intron dots in non-expression regions of the embryo, 2) to minimize the number of false intron dots in the cytoplasm of the embryo, and 3) to minimize the percentage of nuclei that have more than two dots. At our established threshold setting, errors of detected intron dots based on each of these three additional criteria are estimated as follow: 1) using the region of *x*/*L* = 0.55∼0.65, we estimate that there is 0.023 *hb* intron dots per nucleus within the non-expression region (Figure S1B); 2) the ratio of false *hb* intron dots in the cytoplasm to dots inside the nucleus is 0.109 (Figure S1C); and 3) 1.7% of all identified nuclei contain more than 2 *hb* intron dots (Figure S1D). Finally, to further evaluate the performance of our algorithms for automatically identifying intron dots, we compared the machine-recognized intron dots with human-recognized dots and found an uncertainty of 8% using our established threshold setting.

## Supporting Information

Figure S1 **Establishing the threshold setting for detecting intron dots.** (A) Shown is the difference in *hb* intron dot density *ρ* between two expression regions (*x*/*L* = 0.3∼0.4 and *x*/*L* = 0.2∼0.3) as a function of the pixel intensity threshold used in detecting the intron dots. In this test (as well as in tests shown in all the other panels of this figure), the threshold of intron dot size is set at the optimized value of 4 (see Materials and Methods). The dashed line represents the intensity threshold chosen for identifying intron dots analyzed in the current work. (B) Shown is the average number of *hb* intron dots per nucleus as a function of intensity threshold in the non-expression region of *x*/*L* = 0.55∼0.65. (C) Shown is the ratio of identified intron dots outside the nuclear regions to those inside the nuclear regions as a function of intensity threshold. Data shown are extracted from all nuclei identified from 14 embryos. (D) Shown is the percentage of nuclei with more than 2 *hb* intron dots out of all nuclei identified as a function of intensity threshold. Data were extracted from all nuclei identified from 14 embryos.
(TIF)

Figure S2 **Single-embryo analysis.** Using data extracted from single embryos, thus eliminating the between-embryo component from the extrinsic-like fluctuations, we analyzed the enrichment of Bcd concentration within *hb* transcriptionally-active nuclei and at the locations of nascent *hb* transcripts. Shown are two ratio profiles as a function of A-P position at the anterior expression region: the mean Bcd intensity at the intron dot locations over the mean Bcd intensity within active nuclei (red), and the mean Bcd intensity within inactive nuclei over the mean Bcd intensity within active nuclei (blue). Consistent with data from 14 wt embryos, active nuclei generally have more Bcd molecules than inactive nuclei in a single embryo, and the intron dot sites generally have higher Bcd intensities than the environment of the active nuclei in the same embryo.
(TIF)

Figure S3 **Properties of detected** ***otd*** **intron dots.** (A) Shown is a merged image of a wt embryo at early nuclear cycle 14 detecting the nuclear envelope (red), Bcd proteins (blue) and nascent *otd* transcripts as intron dots (green). Shown on the right is a magnified view of a section of the expression region. (B) Shown is the radial distribution of *otd* intron dots. Data shown here were extracted from one single wt embryo with 2,449 identified nuclei (with a mean diameter *l* = 5.88±0.83 µm) and 251 detected intron dots. There are several intron dots that are outside the illustrative mean nuclear boundary since all nuclei are not perfectly round (see Fig. 1 legend for further details). (C) Shown are the measured distances between two detected *otd* intron dots inside individual nuclei. The measured mean distance between two intron dots inside individual nuclei is 2.51±0.29 µm for all 8 tested female embryos (represented by different colors), and each error bar is one standard deviation among the nuclei for a single embryo.
(TIF)
