# Supplementary figures and images for: A Multiscale Investigation of Bicoid-Dependent Transcriptional Events in Drosophila Embryos
Source: PLoS One. 2011 Apr 22;6(4):e19122. doi: 10.1371/journal.pone.0019122 (PMC3081338; doi:10.1371/journal.pone.0019122)

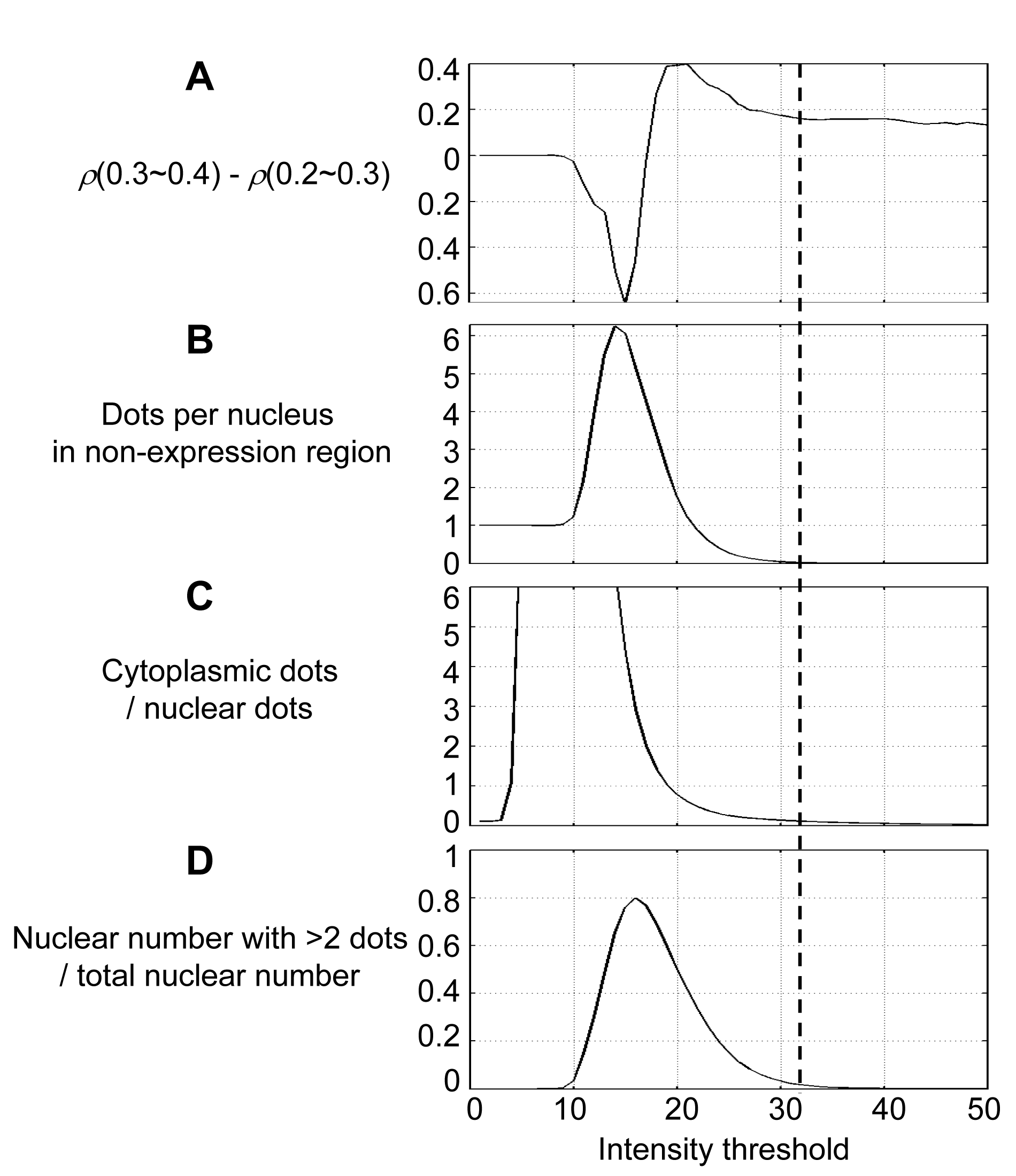

Supplement: Figure S1 — Establishing the threshold setting for detecting intron dots. (A) Shown is the difference in hb intron dot density ρ between two expression regions (x/L = 0.3∼0.4 and x/L = 0.2∼0.3) as a function of the pixel intensity threshold used in detecting the intron dots. In this test (as well as in tests shown in all the other panels of this figure), the threshold of intron dot size is set at the optimized value of 4 (see Materials and Methods). The dashed line represents the intensity threshold chosen for identifying intron dots analyzed in the current work. (B) Shown is the average number of hb intron dots per nucleus as a function of intensity threshold in the non-expression region of x/L = 0.55∼0.65. (C) Shown is the ratio of identified intron dots outside the nuclear regions to those inside the nuclear regions as a function of intensity threshold. Data shown are extracted from all nuclei identified from 14 embryos. (D) Shown is the percentage of nuclei with more than 2 hb intron dots out of all nuclei identified as a function of intensity threshold. Data were extracted from all nuclei identified from 14 embryos. (TIF) [file pone.0019122.s001.tif]

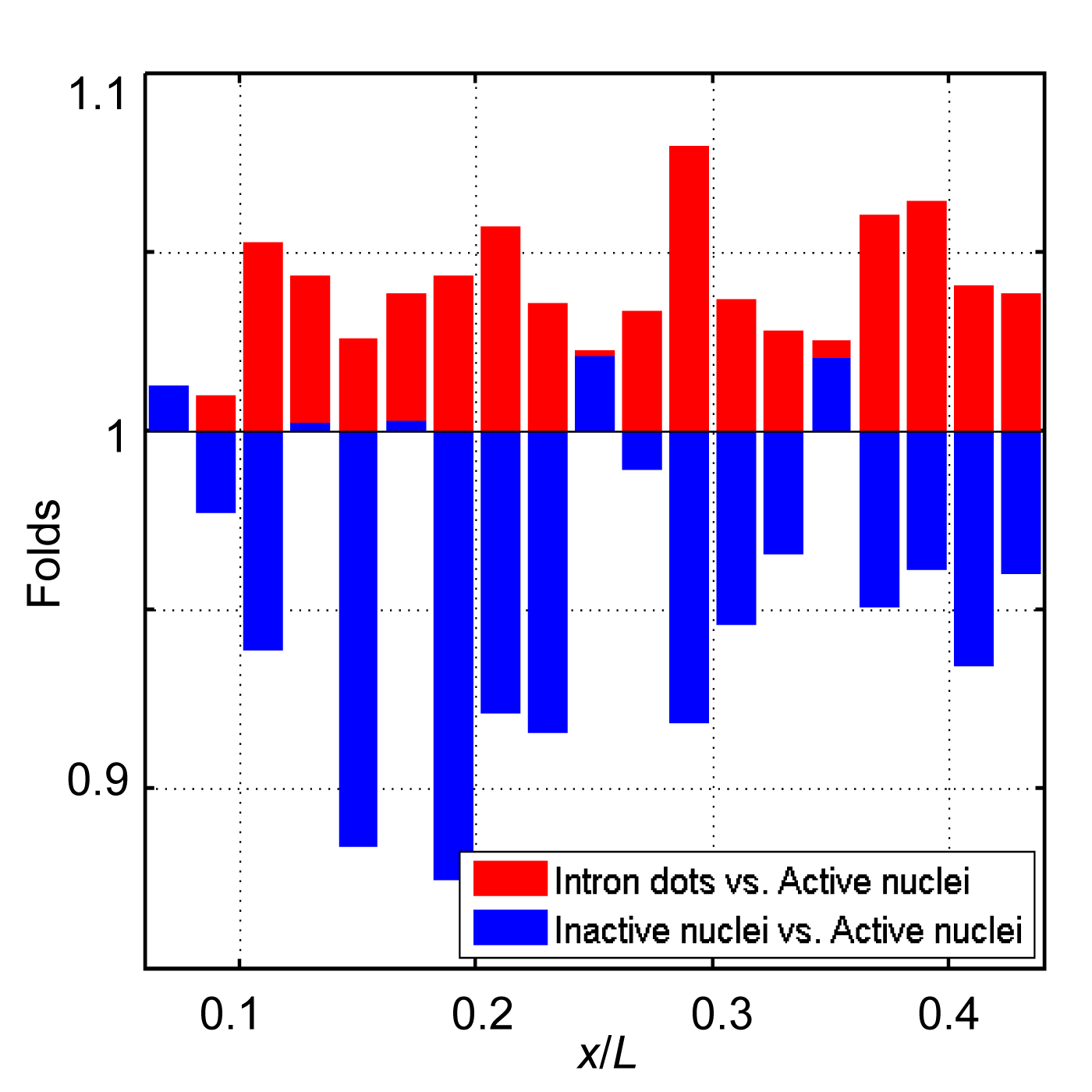

Supplement: Figure S2 — Single-embryo analysis. Using data extracted from single embryos, thus eliminating the between-embryo component from the extrinsic-like fluctuations, we analyzed the enrichment of Bcd concentration within hb transcriptionally-active nuclei and at the locations of nascent hb transcripts. Shown are two ratio profiles as a function of A-P position at the anterior expression region: the mean Bcd intensity at the intron dot locations over the mean Bcd intensity within active nuclei (red), and the mean Bcd intensity within inactive nuclei over the mean Bcd intensity within active nuclei (blue). Consistent with data from 14 wt embryos, active nuclei generally have more Bcd molecules than inactive nuclei in a single embryo, and the intron dot sites generally have higher Bcd intensities than the environment of the active nuclei in the same embryo. (TIF) [file pone.0019122.s002.tif]

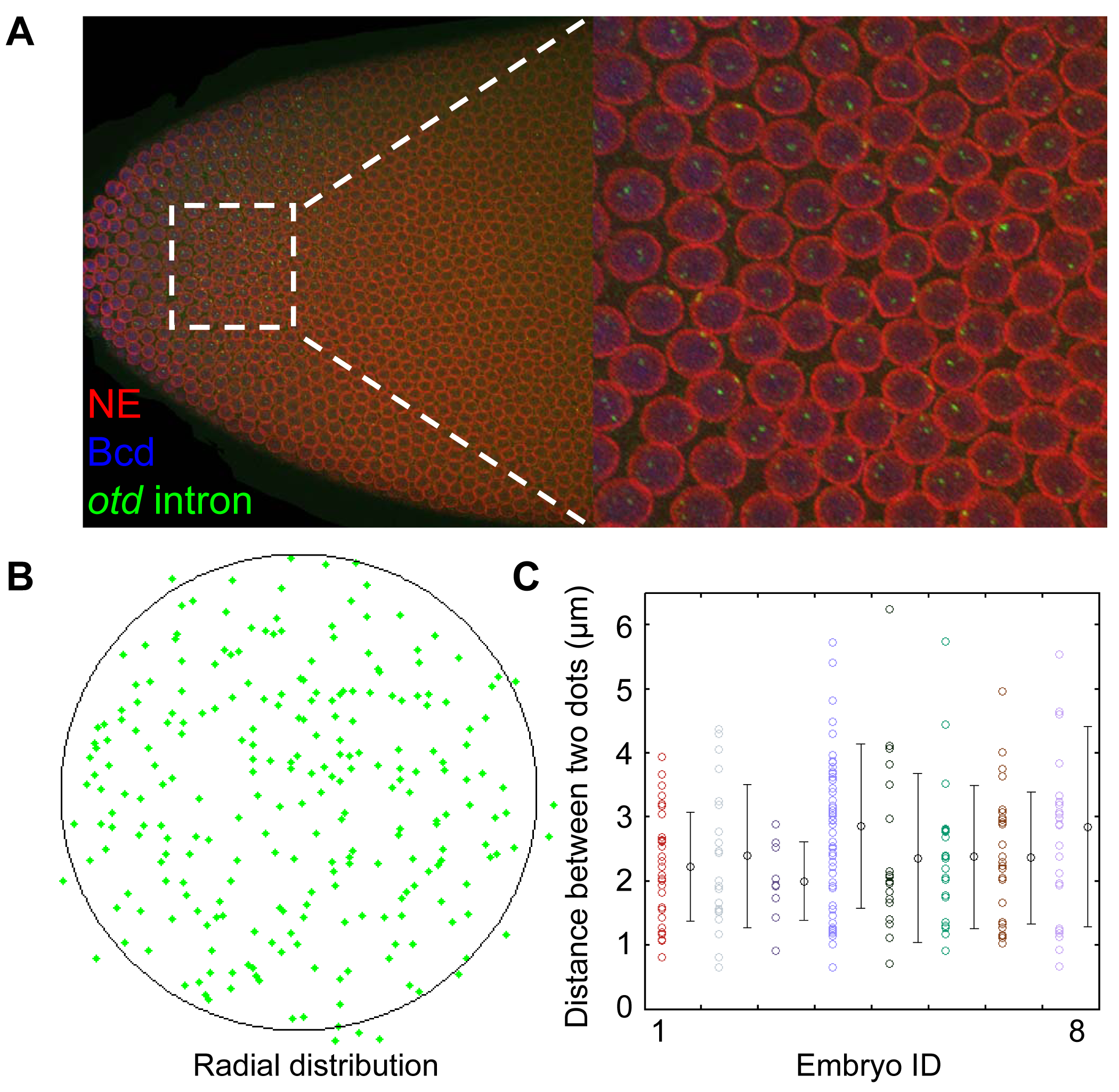

Supplement: Figure S3 — Properties of detected otd intron dots. (A) Shown is a merged image of a wt embryo at early nuclear cycle 14 detecting the nuclear envelope (red), Bcd proteins (blue) and nascent otd transcripts as intron dots (green). Shown on the right is a magnified view of a section of the expression region. (B) Shown is the radial distribution of otd intron dots. Data shown here were extracted from one single wt embryo with 2,449 identified nuclei (with a mean diameter l = 5.88±0.83 µm) and 251 detected intron dots. There are several intron dots that are outside the illustrative mean nuclear boundary since all nuclei are not perfectly round (see Fig. 1 legend for further details). (C) Shown are the measured distances between two detected otd intron dots inside individual nuclei. The measured mean distance between two intron dots inside individual nuclei is 2.51±0.29 µm for all 8 tested female embryos (represented by different colors), and each error bar is one standard deviation among the nuclei for a single embryo. (TIF) [file pone.0019122.s003.tif]
